# Comparative plasma biochemistry analyte data in nesting leatherback (*Dermochelys coriacea*), foraging green (*Chelonia mydas*) and foraging and nesting hawksbill (*Eretmochelys imbricata*) sea turtles in Grenada, West Indies

**DOI:** 10.1093/conphys/coae028

**Published:** 2024-05-17

**Authors:** Madison Kucinick, Kate E Charles, Kenrith Carter, Jonnel Edwards, Catherine Costlow, Melinda Wilkerson, Dawn Seddon, David Marancik

**Affiliations:** Department of Pathobiology, St. George’s University School of Veterinary Medicine, True Blue, Grenada, West Indies; Ocean Spirits, Inc, Levera, Grenada, West Indies; Ocean Spirits, Inc, Levera, Grenada, West Indies; Dr Carter Veterinary Services, St. David's, Grenada, West Indies; Department of Pathobiology, St. George’s University School of Veterinary Medicine, True Blue, Grenada, West Indies; Department of Pathobiology, St. George’s University School of Veterinary Medicine, True Blue, Grenada, West Indies; Department of Pathobiology, St. George’s University School of Veterinary Medicine, True Blue, Grenada, West Indies; Department of Pathobiology, St. George’s University School of Veterinary Medicine, True Blue, Grenada, West Indies; Department of Pathobiology, St. George’s University School of Veterinary Medicine, True Blue, Grenada, West Indies

**Keywords:** Blood, life-stage class, morphometrics, physiology, Abbreviations: AST, aspartate aminotransferase, BMG, bromocresol green, CCL, curved carapace length, CCW, curved carapace width, CK, creatine kinase, GGT, gamma-glutamyl transferase, PIT, passive integrated transponder

## Abstract

Blood biochemistry represents a minimally invasive tool for monitoring sea turtle health, assessing injured sea turtles and supporting conservation strategies. In Grenada, West Indies, plasma biochemical variables were examined in 33 nesting leatherback (*Dermochelys coriacea*), 49 foraging green (*Chelonia mydas*), 49 foraging hawksbill (*Eretmochelys imbricata*) and 12 nesting hawksbill sea turtles sampled between 2017 and 2022. Plasma biochemistry reference intervals are described herein except for nesting hawksbills, which are represented by descriptive statistics due to the low sample size. Select analyte concentrations were positively correlated with curved carapace length in leatherbacks (chloride), green turtles (total protein, albumin and globulin) and foraging hawksbills (total protein, albumin and phosphorus). Cholesterol (7.8 mmol/l ± 1.6 SD) and triglyceride (6.9 mmol/l ± 1.9 SD) concentrations were significantly higher in leatherbacks compared to foraging green turtles, foraging hawksbills and nesting hawksbills (*P* < 0.001 for all). Cholesterol was significantly higher in nesting hawksbills compared to foraging green turtles (*P* = 0.050) and foraging hawksbills (*P* = 0.050). Foraging hawksbills demonstrated significantly higher aspartate transaminase activities than leatherbacks (*P* = 0.002), green turtles (*P* = 0.009) and nesting hawksbills (*P* < 0.001). Biochemical results provide baseline population health data and support guidance for treatments during clinical sea turtle rehabilitation efforts. They also provide insight into species-specific physiologic differences and preludes further studies to better characterize the impacts of life-stage class on biochemistry reference intervals to better support wild sea turtle populations in Grenada.

## Teaser

Plasma biochemistry reference intervals were defined for nesting leatherback, foraging green and foraging hawksbill sea turtles, and descriptive statistics were calculated for nesting hawksbill turtles. Data support population health assessments and management of rehabilitating turtles in Grenada, and interspecies and conspecific comparisons contribute to our understanding of sea turtle physiology.

## Introduction

Grenada is a tri-island nation located within the West Indies consisting of the main islands of Grenada, Carriacou and Petite Martinique. Grenada is part of the Atlantic distribution of nesting leatherback (*Dermochelys coriacea*), foraging (with sporadic nesting) green (*Chelonia mydas*) and foraging and nesting hawksbill (*Eretmochelys imbricata*) sea turtles. Levera Beach on the island of Grenada hosts one of the largest aggregates of nesting leatherbacks in the Caribbean with 250–350 individual turtles nesting annually, although numbers have declined in recent years (Dow *et al.*, 2007; Ocean Spirits, Inc., 2017; Barragan *et al.*, 2022; Charles *et al*., 2022). Resident green turtles and hawksbills are commonly found foraging in the inshore areas of the tri-islands (Grazette *et al.*, 2007). Isle de Caille, one of multiple small, uninhabited islands located between Grenada and Carriacou has been designated as an index nesting grounds in Grenada for hawksbills (Ocean Spirits Inc., 2004).

Leatherback, green and hawksbill turtles are currently listed globally as vulnerable, endangered and critically endangered, respectively, by the International Union for Conservation of Nature (2023). Global populations are under threat due to fishing bycatch, habitat destruction, legal and illegal harvesting, declining water quality and predicted climate change (Jackson *et al.*, 2001; Harris *et al.*, 2018; Blechschmidt *et al.*, 2020). This decline may have implications in the Caribbean region as sea turtles play an important ecologic and economic role throughout the region including balancing marine ecosystems (Bjorndal and Jackson, 2003; Burkholder *et al.*, 2013) and support of ecotourism by attracting customers for scuba diving, snorkeling, nesting sea turtle tours and boat charters (Eckert and Hemphill, 2005).

Assessing and monitoring the health of local sea turtle aggregations is vital to conservation strategies within their broader ranges (Mashkour *et al.*, 2020). Plasma biochemical analytes have been used to characterize the health of numerous free-ranging sea turtle species in nesting and foraging habitats (Bolten and Bjorndal, 1992; Aguirre and Balazs, 2000; Deem *et al.*, 2009; Anderson *et al.*, 2011; Harris *et al.*, 2011; Ehsanpour *et al.*, 2015; Muñoz-Pérez *et al.*, 2017; Espinoza-Romo *et al.*, 2018; Fernández-Sanz *et al.*, 2023; Stacy *et al.*, 2023). Key plasma biochemical analytes have been shown to have diagnostic and prognostic value in sea turtles affected by infection, injury, starvation, dehydration (Aguirre and Balazs, 2000; Lopez-Mendilaharsu *et al.*, 2005; Deem *et al.*, 2009; Hayakijkosol *et al.*, 2024) and anthropogenic pollutants (Camacho *et al.*, 2013; Espinoza-Romo *et al.*, 2018). This assessment technique has also been used to determine the efficacy of captive diets in meeting the nutritional requirements of rehabilitating green turtles compared to wild populations (Stringer *et al.*, 2010).

In ectotherms, the adaptation to maintain homeostasis at varying temperatures can complicate the interpretation of plasma biochemical results. Variations in biochemical analytes can be physiologic in healthy reptiles due to different metabolic capacities, whereas these variations may indicate disease in other vertebrates (Bullock, 1955; Khaki *et al.*, 2024). Sea turtle biochemical analytes may be further affected by their migratory life cycles, where regional characteristics such as life-stage class, diet, reproductive status, size and water quality impact their physiology (Deem *et al.*, 2009; Stacy *et al.*, 2023). For example, serum triglycerides and cholesterol concentrations have been found to be higher in nesting loggerhead sea turtles (*Caretta caretta*) compared to foraging or stranded conspecifics, corresponding to the process of egg production (Deem *et al.*, 2009). Studies report that total protein concentrations are strongly correlated with size of green turtles, while uric acid and cholesterol concentrations have been shown to significantly differ between sexes (Bolten and Bjorndal, 1992). Species variation has been reported in post-prandial changes of total protein and cholesterol concentrations and aspartate aminotransferase (AST) activities between green turtles and Kemp’s ridley sea turtles (*Lepidochelys kempii*), suggesting that natural differences in diet or species-specific metabolism may impact biochemical results (Anderson *et al.,* 2011). Varied results have also been recorded for the same sample across different biochemistry analyzers highlighting the effect of testing methods and technologies on quantifying analyte concentrations (Wolf *et al.*, 2008). Therefore, the integrity of biochemistry interpretation relies on the development of location-specific, and ideally age and sex-specific, analyte reference intervals in each sea turtle species within individual laboratories.

The current study aimed to examine the physiologic health and establish plasma biochemistry reference intervals for select plasma analytes for nesting leatherback and foraging green turtle and hawksbill aggregations in Grenada. Physiologic health was evaluated in a relatively smaller cohort of nesting hawksbills as limited sample numbers did not provide a statistically significant sample size (*n* = 12) to establish reference intervals (Friedrichs *et al*., 2012). This study compared the means of each analyte across the three species found within the same habitat and examined associations between analytes and carapace length. The purpose of this comparative study of plasma biochemical analytes was to strengthen the sea turtle health assessment programme on the island, enhance the treatment of turtles admitted to rehabilitation facilities, aid in monitoring population health changes over time and provide baseline comparisons between both heterospecific and conspecific populations worldwide.

## Material and Methods

The protocols and procedures of this study were approved by the St. George’s University Institutional Animal Care and Use Committee (IACUC-16017-R). Research permits were obtained from the Grenada Ministry of Agriculture, Forestry, Lands and Fisheries.

### Leatherback sampling

**Figure 1:**
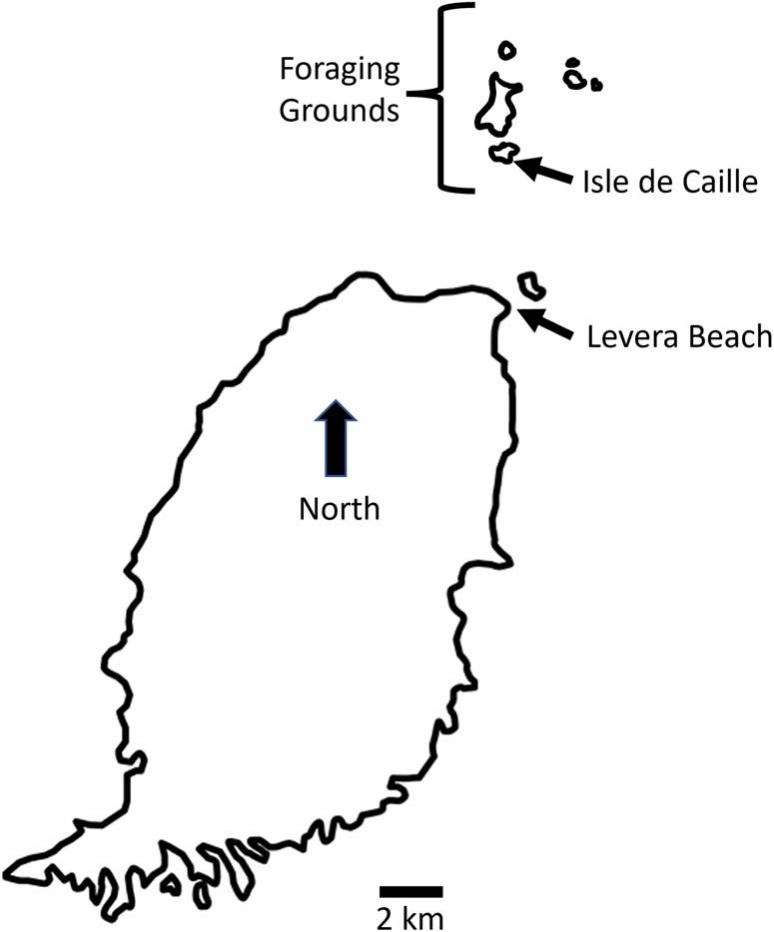
Map of Grenada depicting sampling sites for nesting leatherback turtles (*Dermochelys coriacea*) on Levera Beach, green (*Chelonia mydas*) and hawksbill (*Eretmochelys imbricata*) turtles within inshore foraging grounds, and nesting hawksbill turtles on Isle de Caille.

Blood samples were collected from nesting leatherbacks from March through July 2017–2019 on Levera Beach, Grenada (Fig. 1). Leatherbacks were sampled only once during nesting season when first encountered on the beach. All turtles included in this study were examined and interpreted as apparently healthy, as defined by subjective assessment of adequate body condition and no external lesions or obvious signs of disease present. Turtles were approached during ovipositioning for assessment and blood sampling and not manually restrained. Carapace measurements were taken from the nuchal notch to the terminal tip of the caudal peduncle along the top ridge for curved carapace length (CCLmax) and at the widest region of the carapace for curved carapace width (CCW). Monel flipper tags with identification numbers were placed or recorded, if present, and passive integrated transponder (PIT) tags were additionally placed in the left shoulder per standard procedures or recorded, if present (Eckert and Beggs, 2006). Venipuncture was performed from the interdigital vein during ovipositioning. The venipuncture site was disinfected with 5% betadine solution prior to and directly after blood collection. A 22-gauge, 1-inch needle and 3 ml syringe (Kendall Monoject, Mansfield, MA) were used to collect up to 3 ml of blood. The blood was transferred into a lithium heparin tube (Greiner Bio-One, Monroe, NC, USA) and placed in bags insulated with paper towels and placed on ice until transportation to the laboratory within 6 hours.

### Foraging green turtle and hawksbill sampling

Blood samples were collected from foraging green turtles and hawksbills from June through August 2017–2022 in the offshore waters north of Grenada (Fig. 1). Turtles were captured by freedivers and immediately transferred to a boat. Each turtle was measured for curved carapace length from notch to tip (CCL-nt) and CCW, given a visual health assessment, and Inconel flipper tags were placed and/or recorded bilaterally (Eckert and Beggs, 2006). Life-stage class for green turtles was estimated from carapace length with juveniles < 65 cm CCL, subadults 65–90 cm CCL and adults > 90 cm CCL (Wyneken, 2003). Life-stage class for foraging hawksbills is not as well defined and was conservatively estimated as being adult at > 75 cm CCL (Moncada *et al.,* 1999). Relative tail length and girth were used to sex green turtles and hawksbills that demonstrated sex dimorphism and trended towards larger subadult or adult CCL. Sexing was not attempted in juvenile or smaller subadult turtles due to lack of sex dimorphic traits.

Each turtle was manually restrained in a prone position, with the head pointing down and the neck extended using gentle traction. One millilitre of blood was collected from the external jugular vein in immature turtles and up to 3 ml of blood was collected from subadult and adult turtles, and the venipuncture site was disinfected with 5% betadine solution prior to and immediately following sampling. Blood samples were stored and transported as described for leatherbacks.

### Nesting hawksbill sampling

Nesting hawksbills on Isle de Caille were sampled in June through August 2022 (Fig. 1). Turtles were approached during ovipositioning and not manually restrained except for repositioning of the head and neck to provide access to the external jugular vein for venipuncture. Each turtle was given a visual health assessment and measured for CCLn-t and CCW. Inconel flipper tags were placed and/or recorded bilaterally. Up to 3 ml of blood was collected from the cervical jugular vein using aseptic technique, and blood was stored and transported as described above.

### Sample processing and analysis

Whole blood was centrifuged at 1008 relative centrifugal force for 10 minutes within 6 hours of collection, and plasma was removed and stored at −80°C for up to 1 year prior to analysis with no freeze–thaw cycles. Plasma potassium, sodium, chloride and lipaemia, hemolysis and icterus were quantified (0 = absent, 1 = mild, 2 = moderate, 3 = severe) using the Abaxis VetScan VS2 Chemistry Analyzer (Zoetis, New Jersey, USA). An IDEXX VetTest Chemistry Analyzer (IDEXX Laboratories, Maine, USA) was used to quantify plasma total protein, triglycerides, cholesterol, glucose, calcium, phosphorus, creatine kinase (CK), AST, gamma-glutamyl transferase (GGT) and uric acid. Quantified albumin concentrations (IDEXX VetTest Chemistry Analyzer) were used as an estimation of this analyte as the bromocresol green (BMG) biochemistry technology utilized by the VetTest Analyzer has been shown to weakly correlate with albumin concentrations when compared to gel electrophoresis quantification (Muller and Brunnberg, 2010; Macrelli *et al.,* 2013). Subsequently, globulins were also estimated by subtracting the albumin concentration from the total protein concentration. Analyte concentrations and enzyme activities were converted from conventional to international (IS) units using a standardized conversion table (https://diagnostics.be/conversiontable_EN).

### Statistical analysis

Leatherback, green turtle and hawksbill plasma analyte concentrations were examined following the guidelines of the American Society of Veterinary Clinical Pathology (Friedrichs, et al., 2012) using the algorithms in Reference Interval Advisor 2.1 (Geffré *et al.*, 2011). Descriptive statistics including analyte minimum, maximum, mean and median values were calculated for each species. The distribution of the data was determined to be Gaussian (normal) or non-Gaussian by histogram and Kolmogorov–Smirnov test. Normality results are reported in Tables 1–3. Potential outliers were examined using the Dixon’s range statistic method. Data identified as potential outliers were examined graphically and within the context of clinical observations to determine whether to include those data points. The 90% confidence intervals were determined by a non-parametric bootstrap method following Box–Cox transformation for Gaussian distributed data, and non-Gaussian distributed data were examined using the robust method following Box–Cox transformation.

**Table 1:** Plasma biochemical analyte descriptive statistics and reference intervals for leatherback sea turtles (*Dermochelys coriacea*) in Grenada, West Indies^a^

Analyte	*N*	Min	Max	Median	Mean	Lower limit (90% CI)	Upper limit (90% CI)
Total protein (g/l)^b^	33	20	52	45	43	27 (18, 34)	52 (51, 54)
Albumin (g/l)^b^	33	6	22	15	15	7 (4, 9)	23 (21, 24)
Globulin (g/l)^b^	33	14	38	29	28	16 (1, 21)	37 (35, 38)
Glucose (mmol/l)^b^	33	2.7	5.3	4.4	4.3	3.2 (2.7, 3.5)	5.2 (5.1, 5.3)
Cholesterol (mmol/l)^b^	33	2.6	11.7	7.7	7.8	4.0 (3.1, 5.0)	10.8 (10.0, 11.6)
Triglycerides (mmol/l)^b^	33	3.4	10.4	6.9	6.9	2.9 (2.0, 4.1)	10.8 (9.7, 11.7)
Uric acid (mmol/l)^b^	33	<0.001	0.004	-	-	-	-
AST (μkat/l)^b^	33	1.71	5.59	2.59	2.71	1.76 (1.61, 196)	4.15 (3.60, 4.80)
GGT (μkat/l)	33	0.00	0.05	0.00	0.01	0.00 (0.00,0.00)	0.05 (0.03, 0.07)
Creatine kinase (μkat/l)	33	0	33.84	6.95	8.85	0.01 (0, 0..06)	35.69 (26.57, 45.16)
Calcium (mmol/l)^b^	33	1.50	2.85	2.33	2.38	1.68 (1.50, 1.85)	2.88 (2.78, 2.95)
Phosphorus (mmol/l)^b^	33	3.26	4.68	4.13	4.10	3.29 (3.07, 3.52)	4.75 (4.59, 4.88)
Sodium (mmol/l)^b^	33	130	156	141	141	132 (130, 134)	153 (150, 157)
Potassium (mmol/l)^b^	33	3.7	6.5	4.5	4.6	3.7 (3.5, 3.8)	6.0 (5.6, 6.5)
Chloride (mmol/l)^b^	33	98	113	107	107	98 (95, 100)	114 (112, 115)

a ^a^Plasma uric acid was below the detection limit of the analyzer in 29/33 samples, and no reference intervals are provided.

b ^b^Analytes with normal distribution. AST: aspartate transaminase; GGT: gamma-glutamyl transferase.

Plasma analyte concentrations and enzyme activities were compared to hemolysis, lipemia and icterus scores using a one-way analysis of variance (ANOVA). Calculated analyte means were compared between each of the three species (foraging and nesting hawksbills were included separately) using a two-way ANOVA to examine interspecies variations and variations between foraging and nesting hawksbills. A two-way ANOVA was utilized to examine analyte differences between sex and life-stage class for hawksbills only. Analyte results were evaluated in relation to host CCL-nt and CCW using linear regression and a test of significance to examine their correlation with turtle size. No statistical differences were seen in analyte correlation when using CCL-nt versus CCW and the former was used as a surrogate representative of turtle size. All statistical analysis was performed using Graph Pad Prism 8 with a significance level of *p* ≤ 0.050.

## Results

All turtles were assessed to be alert with no signs of significant external injuries or tumors and with minimal barnacle or algal growth on the carapace. Observed lesions that were interpreted to have minimal effect on physiologic health included healed soft tissue injuries to the neck or flippers, missing sections of flippers, scratched carapace and missing or damaged marginal scutes in the carapace. A total of 33 nesting leatherbacks (CCL 147.0 cm ± 5.1 SD, CCW: 110.0 cm ± 10.1 SD), 49 green turtles (one adult female, two subadult, 46 juvenile, CCL-nt: 47.3 cm ± 11.2 SD, CCW: 42.7 cm ± 10.9 SD), 49 foraging hawksbills (21 females, five males, 23 juvenile/subadult, CCL-nt: 66.0 cm ± 21.9 SD, CCW: 60.4 ± 20.1 SD) and 12 nesting hawksbills (CCL-nt: 85.7 cm ± 5.0 SD, CCW: 77.1 cm ± 4.0 SD) were included in this study.

Initially, 42 nesting leatherbacks were sampled. Five turtles with moderate to severe lipemia were excluded from analysis as lipaemic index demonstrated a significant association with total protein (*P* < 0.001), albumin (*P* = 0.002), triglyceride (*P* < 0.001) and calcium (*P* < 0.001). Four additional samples with moderate hemolysis were excluded from analysis. Of the 54 plasma samples collected for green turtles, 5 samples were excluded due to moderate hemolysis. Fifty-five foraging hawksbills and 14 nesting hawksbills were originally sampled, but 2 foraging hawksbill and 2 nesting hawksbill samples were excluded from analysis due to consistent outlier observations in greater than 50% of the analytes examined and relatively dilute blood noted on venipuncture. An additional four foraging hawksbills were excluded due to moderate plasma hemolysis. When leatherback, green turtle and foraging hawksbill samples with moderate and severe hemolysis were included in the analysis. There was no significant association of hemolytic index with analyte concentrations or enzyme activities. However, these were excluded out of caution due to possible effects on plasma analyte quantification (Simundic *et al*., 2020; Stacy *et al.*, 2019).

### Leatherbacks

**Figure 2:**
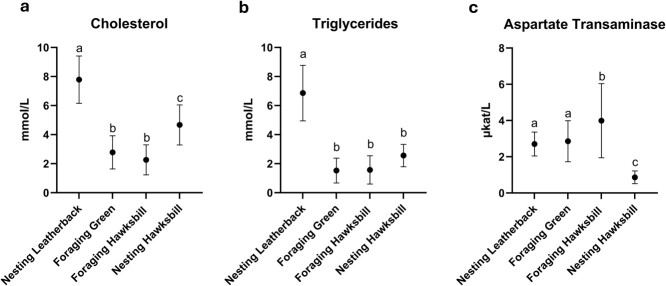
Mean and standard deviation for plasma cholesterol and triglycerides concentrations and aspartate transaminase activity that significantly differed between nesting leatherback (*Dermochelys coriacea*), foraging green (*Chelonia mydas*) and foraging and nesting hawksbill (*Eretmochelys imbricata*) sea turtles. Statistical groupings are notated by letter.

Descriptive statistics, reported upper and lower limits of the reference intervals and corresponding 90% confidence intervals for 14/15 analytes are described in Table 1. Uric acid was below the detection limit (<0.001 mmol/l) of the VetTest analyzer for 29/33 (87.9%) samples, and the minimum and maximum concentrations are provided (Table 1). There was low hemolysis in 19/33 (57.6%) and low lipemia in 20/33 (60.6%) samples without significant associations with analyte concentrations. All icterus scores were zero. There was a moderate, positive correlation between CCLmax and chloride (*r* = 0.46, *P* = 0.020). Plasma cholesterol (7.8 mmol/l ± 1.6 SD) and triglyceride (6.9 mmol/l ± 1.9 SD) were significantly higher in leatherbacks (*P* < 0.001 for all) compared to green turtles (cholesterol: 2.8 mmol/l ± 1.1 SD; triglycerides: 1.5 mmol/l ± 0.8 SD), foraging hawksbills (cholesterol: 2.3 mmol/l ± 1.0 SD; triglycerides: 1.6 mmol/l ± 1.0 SD), and nesting hawksbills (cholesterol: 4.7 mmol/l ± 1.3 SD; triglycerides: 2.5 mmol/l ± 0.7 SD) (Fig. 2a and b).

**Table 2:** Plasma biochemical analyte descriptive statistics and reference intervals for green sea turtles (*Chelonia mydas*) in Grenada, West Indies^a^

Analyte	*N*	Min	Max	Median	Mean	Lower limit (90% CI)	Upper limit (90% CI)
Total protein (g/l)^b^	49	26	62	39	42	28 (26, 30)	58 (53, 62)
Albumin (g/l)^b^	49	7	21	13	13	8 (8, 9)	1.8 (17, 20)
Globulin (g/l)^b^	49	17	44	27.5	28	19 (17, 20)	41 (37, 43)
Glucose (mmol/l)^b^	49	1.1	5.1	3.1	3.2	1.7 (1.3, 1.9)	4.8 (4.4, 5.2)
Cholesterol (mmol/l)^b^	49	0.4	5.4	2.9	2.9	1.1 (0.8, 1.5)	4.6 (4.2, 5.0)
Triglycerides (mmol/l)^b^	49	0.0	4.2	1.5	1.6	04 (0.2, 0.6)	3.1 (2.7, 3.5)
Uric acid (mmol/l)^b^	49	<0.001	0.08	-	-	-	-
AST (μkat/l)^b^	49	0.00	4.98	2.86	2.97	0.32 (0.98, 1.68)	4.81 (4.33, 5.26)
GGT (μkat/l)	49	0.00	0.18	0.00	0.02	0.00 (0.00,0.00)	0.17 (0.07, 0.18)
Creatine kinase (μkat/l)^b^	49	0	18.60	8.45	9.15	2.33 (1.48, 3.28)	17.85 (15.09, 20.47)
Calcium (mmol/l)^b^	49	1.15	3.08	2.15	2.13	1.48 (1.33, 1.63)	3.78 (2.65, 2.90)
Phosphorus (mmol/l)^b^	49	0.23	4.07	2.16	2.16	1.16 (0.90, 1.42)	3.07 (2.81, 2.33)
Sodium (mmol/l)	49	130	156	141	141	145 (143, 146)	157 (155, 158)
Potassium (mmol/l)^b^	49	3.7	6.5	4.5	4.6	4.9 (4.8, 5.2)	6.7 (6.5, 6.8)
Chloride (mmol/l)^b^	49	98	113	107	107	104 (902, 106)	119 (117122)

a ^a^Plasma uric acid was below the limit of the analyzer in 16/49 samples, and no reference intervals are provided.

b ^b^Analytes with normal distribution. AST: aspartate transaminase; GGT: gamma-glutamyl transferase.

### Green turtles

The descriptive statistics, upper and lower limits of the reference intervals and corresponding 90% confidence intervals are described in Table 2. For uric acid, 16/49 samples (32.7%) were below the detection limit of the VetTest analyzer (<0.001 mmol/L) and only the range is provided (Table 1). Hemolysis was low in 13/49 (26.5%) of samples without significant association between hemolytic index and analyte concentrations and enzyme activities. Lipemia and icterus were absent. CCL-nt had a moderate, positive correlation with total protein (*r* = 0.48, *P* = 0.002) and globulin (*r* = 0.49, *P* = 0.010) and low, positive correlation with albumin (*r* = 0.33, *P* = 0.040) concentrations. There were no significant differences in analyte results between juveniles and subadults.

### Foraging hawksbills

The descriptive statistics, upper and lower limits of the reference intervals and corresponding 90% confidence intervals are described in Table 3. Hemolysis was low (14/49, 28.6%) and not significantly associated with analyte concentrations. Lipemia and icterus were absent. CCL-nt was strongly and positively correlated with total protein (*r* = 0.69, *P* < 0.001), albumin (*r* = 0.70, *P* < 0.001) and phosphorus (*r* = 0.62, *P* < 0.001) and weakly positively correlated with calcium concentrations (*r* = 0.36, *P* = 0.03). Foraging hawksbills demonstrated significantly higher AST activities (3.99 μkat/l ± 2.03 SD) than leatherbacks (2.71 μkat/l ± 0.65 SD, *P* = 0.002), green turtles (2.86 μkat/l ± 1.12 SD, *P* = 0.009) and nesting hawksbills (0.86 μkat/l ± 0.33 SD, *P* < 0.0001) (Fig. 2c).

### Nesting hawksbills

Descriptive statistics are reported in Table 4 as the sample size did not reach statistical significance to allow development of 90% confidence intervals. Hemolysis was low in all samples and not significantly associated with analyte concentrations, while lipemia and icterus were absent. No significant correlations were observed between plasma analytes and CCL n-t. Nesting hawksbills demonstrated significantly higher cholesterol concentrations than foraging green turtles (*P* = 0.050) and foraging hawksbills (*P* = 0.050) and significantly lower cholesterol concentrations than nesting leatherbacks (*P* = 0.007) (Fig. 2a). Aspartate transaminase activities were significantly lower than nesting leatherbacks (*P* = 0.020), foraging green turtles (*P* = 0.004) and foraging hawksbills (*P* < 0.001) (Fig. 2c).

## Discussion

The goal of this study was to assess overall physiologic health and define baseline plasma biochemical health variables in nesting leatherback, foraging green turtle and foraging and nesting hawksbill aggregations in Grenada. This is the first study to characterize plasma biochemistry of these species in Grenada and provides the means to examine inter- and intra-species variations in biochemical characteristics within the same geographic region and with consistent analytical methodology. Established reference intervals provide baseline data to monitor population health in nesting and foraging aggregations and for clinically assessing stranded and rehabilitating sea turtles in Grenada. Although foraging turtles were sampled during a three-month period each year, surface water temperatures in Grenada are relatively stable with seasonal variations ranging between 27.1 and 29.4°C (seatemperature.org). This may reduce seasonal temperature effects on metabolism and activity seen in other regions that can alter analyte concentrations and enzymatic activities and thus require seasonal reference intervals (Stacy *et al.*, 2023).

All included study turtles were interpreted on examination to be in good physical health and the plasma biochemical results supported this finding. Blood analyte results appear to be in general agreement with those described from comparable sample populations from other regions, although data comparison is limited by differences in analyzer methodology, analytes examined and data presentation. When examining trends, there are select analytes that appear to show some differences between sea turtle data compiled in Grenada compared with previously published data from other regions. Leatherbacks sampled in Grenada demonstrated relatively lower median calcium concentrations at 2.38 mmol/l compared to nesting leatherbacks compiled from Papua New Guinea (*n* = 11), Costa Rica (*n* = 8) and St. Croix (*n* = 12) (Harris *et al.*, 2011), Florida, USA (Perrault *et al.*, 2012) and from the Gulf of Guinea, Africa (Honarvar *et al.*, 2011) at 2.75, 2.63 and 2.90 mmol/l, respectively. This trend was not uniform for all studies as plasma calcium concentrations in Grenada trended slightly higher than the median 2.2 and 2.3 mmol/l described for leatherbacks nesting in Florida (Stacy *et al.*, 2019) and St. Kitts (Stewart *et al.*, 2023), respectively. The 46 g/l median total protein concentration in leatherbacks in Grenada trended higher than a 38 g/l median for turtles sampled in Florida (Stacy *et al.*, 2019) and 39 g/l mean for turtles sampled in St. Kitts (Stewart *et al.*, 2023). Total protein was similar to 48 g/l described in a separate study of nesting leatherbacks in Florida (Perrault *et al.*, 2012) and 49.6 g/dl in St. Croix, Virgin Islands (Perrault *et al.*, 2014), with the latter quantified using gel electrophoresis. Additionally median plasma cholesterol of 7.8 mmol/l in Grenada trended lower than compiled median concentration of 9.1 mmol/l from Papua New, Guinea, Costa Rica and St. Croix (Harris *et al.*, 2011) and mean 9.25 mmol/l from Florida (Perrault *et al.*, 2012). These differences for calcium, total protein and cholesterol were relatively small and may be associated with regional environmental impacts on metabolism including water temperature and diet (Deem *et al.*, 2009; Osborne *et al.*, 2010) and/or migration distances. It is unknown whether leatherbacks nesting in Grenada share foraging grounds and migration routes with the aggregations described above as the life history and haplotype data of leatherbacks nesting in Grenada is largely unknown. Previous studies utilizing satellite transmitters have shown leatherbacks nesting in Trinidad, Suriname and French Guinea utilize foraging grounds comprising the wider Atlantic Ocean (Turtle Expert Working Group, 2007) while haplotype data reveal foraging leatherbacks off the southeastern coast of South America originate from rookeries in West Africa (Vargas *et al.*, 2008; Dutton *et al.*, 2013). Limited satellite tagging data for leatherbacks in Grenada demonstrates some individuals migrate from Atlantic Canada (Fisheries and Oceans Canada, 2022) but more in-depth haplotype characterization is needed to characterize this population.

**Table 3:** Plasma biochemical analyte descriptive statistics and reference intervals for foraging hawksbill sea turtles (*Eretmochelys imbricata*) in Grenada, West Indies

Analyte	*N*	Min	Max	Median	Mean	Lower limit (90% CI)	Upper limit (90% CI)
Total protein (g/l)^a^	49	15	64	38	37	15 (15, 18)	61 (52, 64)
Albumin (g/l)^a^	49	5	.3	.5	.5	7 (5, 10)	22 (19, 23)
Globulin (g/l)^a^	49	02	47	24	22	3 (2, 5)	44 (35, 47)
Glucose (mmol/l)^a^	49	2.9	6.6	4.9	4.8	3.0 (2.9, 3.4)	6.5 (5.8, 6.6)
Cholesterol (mmol/l)^a^	49	0.6	4.6	2.2	2.2	0.6 (0.6, 0.9)	4.6 (4.2, 4.6)
Triglycerides (mmol/l)^a^	49	0.3	4.2	1.3	1.6	0.0 (0.3, 0.7)	4.2 (4.2, 4.2)
Uric acid (mmol/l)^a^	49	0.01	0.06	0.03	0.03	0.01 (0.00, 0.01)	0.06 (0.05, 0.06)
AST (μkat/l)	49	0.91	11.14	3.78	3.98	0.90 (0.66, 1.28)	8.95 (7.72, 10.23)
GGT (μkat/l)	49	0.00	1.33	0.02	0.08	0.00 (0.00,0.00)	1.00 (0.22, 1.33)
Creatine kinase (μkat/l)^a^	49	0	22.79	8.30	9.74	0.22 (0.00, 1.30)	22.59 (21.09, 22.79)
Calcium (mmol/l)^a^	49	1.48	3.43	2.30	2.28	1.50 (1.48, 1.65)	3.28 (2.80, 3.43)
Phosphorus (mmol/l)	49	1.49	2.58	2.13	2.13	1.50 (1.49, 1.74)	3.28 (2.80, 3.43)
Sodium (mmol/l)	49	134	163	150	150	136 (134, 143)	162 (157, 163)
Potassium (mmol/l)	49	3.5	6.4	5.0	5.0	3.5 (3.5, 3.9)	6.4 (6.1, 6.4)
Chloride (mmol/l)	49	100	127	111	113	101 (100, 103)	126 (122, 127)

a ^a^Analytes with normal distribution. AST: aspartate transaminase; GGT: gamma-glutamyl transferase.

**Table 4:** Plasma biochemical analyte ranges, median, and mean for nesting hawksbill sea turtles (*Eretmochelys imbricata*) in Grenada, West Indies

Analyte	*N*	Min	Max	Median	Mean
Total protein (g/l)^a^	12	18	77	42	45
Albumin (g/l)^a^	12	4	30	16	17
Globulin (g/l)^a^	12	14	57	30	31
Glucose (mmol/l)^a^	12	3.9	6.9	5.3	5.3
Cholesterol (mmol/l)^a^	12	2.1	7.2	2.2	4.7
Triglycerides (mmol/l)^a^	12	1.4	3.8	1.6	2.5
Uric acid (mmol/l)^a^	12	0.01	0.07	0.03	0.04
AST (μkat/l)^a^	12	0.33	1.41	3.98	0.86
GGT (μkat/l)	12	0.00	0.30	0.08	0.08
Creatine kinase (μkat/l)^a^	12	0.92	33.94	9.74	13.30
Calcium (mmol/l)^a^	12	1.78	4.00	2.28	2.90
Phosphorus (mmol/l)^a^	12	1.55	4.49	2.13	2.97
Sodium (mmol/l)^a^	12	18	77	42	45
Potassium (mmol/l)^a^	12	4	30	16	17
Chloride (mmol/l)	12	14	57	30	31

a ^a^Analytes with normal distribution. AST: aspartate transaminase; GGT: gamma-glutamyl transferase.

Plasma biochemical results may also be impacted by inter-nesting intervals in nesting turtles. Each leatherback in this study was sampled once from April through July, which likely includes samples taken during different nesting episodes as leatherbacks nest multiple times per year (Girondot *et al.*, 2006). Turtles sampled later in the nesting season are likely affected by extended physiologic influences of hyporexia and egg production (Honarvar *et al.*, 2011; Plot *et al.*, 2013; Perrault *et al.*, 2014; Price *et al.*, 2019; Stewart *et al.*, 2023). Both cholesterol and triglycerides have been shown to increase during vitellogenesis and along with total protein, conversely decrease with anorexia and malnutrition (Kerr, 2002) as extended fasting is observed in nesting sea turtles (Anderson *et al.*, 2011). Mobilization of lipids was also evident in the five plasma samples that had moderate or severe lipemia. As this was significantly associated with total protein, albumin, calcium and triglyceride concentrations, they were excluded from analysis due to possible interference with analyte quantification. Lipemia can be removed from samples using high speed or ultracentrifugation (Castro-Castro *et al*., 2018) although this process requires advanced equipment that may not be present in many laboratories and is not conducive to processing samples in the field.

Relatively higher concentrations of total calcium have been described in nesting sea turtles compared to non-nesting conspecifics due to elevated protein-bound calcium during follicular development (Mader, 2005; Casal *et al.*, 2009). Based on mammalian physiology, 40% of protein-bound calcium is associated with albumin (Clase *et al.*, 2000) and we were not able to accurately interpret this relationship due to overestimation of albumin concentrations using the BMG method for quantification (Muller and Brunnberg, 2010; Macrelli *et al.*, 2013). Repeat sampling throughout nesting season is needed to examine these effects on the physiology and subsequent biochemistry reference intervals in leatherbacks in Grenada. Additionally, the majority of leatherbacks in this study were sampled in 2017 with three turtles sampled in 2018 and two turtles sampled in 2019. Increased sample sizes in subsequent years would help reveal changes in blood values across nesting season for this population.

Foraging green turtles also showed slight variations in total protein and cholesterol concentrations compared to turtles sampled from other locations. This similarly may be associated with regional dietary and temperature differences that can affect metabolic processes (Putillo *et al.*, 2020; Melvin *et al.*, 2022). Green turtles are primarily herbivorous and diets can vary by region based on availability of food items including sea grass and algae (Garnett *et al.*, 1985; Sarkis *et al.*, 2022). The 42 g/l mean total protein concentration in green turtles in Grenada trended between published means of 51 g/l (Bolten and Bjorndal, 1992) and 31 and 38 g/l (Putillo *et al.*, 2020) described for green turtles in the Bahamas at different foraging sites. The mean protein concentrations were also higher than mean 38 g/l described in turtles from the Bahamas (Stewart *et al.*, 2016) and the median 39 g/l total protein was slightly higher than the median 30 g/l described for green turtles in North Carolina, USA (Anderson *et al.*, 2011). The mean cholesterol concentration of 2.9 mmol/l was lower than the mean of 5.6 mmol/l described for green turtles in the Bahamas (Bolten and Bjorndal, 1992) and likewise, the 5.6 mmol/l upper reference interval described for cholesterol in green turtles in Puerto Rico was higher than those calculated for Grenada at 4.6 mmol/l (Page-Karjian *et al*., 2014). As these differences in plasma analyte concentrations between other regions were comparable and likely associated with dietary influences, foraging green turtles in Grenada were considered clinically healthy.

The 47.3 cm mean CCLn-t for green turtles trended towards carapace length measurements commonly described for juvenile green turtles (Wyneken, 2003; Foley *et al.*, 2005; Meylan *et al.*, 2011) indicating a high proportion of immature turtles in this study. However, life-stage class characterization of the sample population is hindered by a lack of regional growth curve data to identify the size at which green turtles reach maturity in Grenada. There was a moderate, positive correlation observed between total protein and carapace size suggesting that life-stage class may influence plasma total protein concentrations. This is consistent with previous studies in green turtles (Bolten and Bjorndal, 1992; Stewart *et al.*, 2016; Stacy and Innis, 2017) and total protein has been shown to increase with carapace size in most sea turtle species (Frair and Shah, 1982). This is likely associated with higher metabolic growth requirements in juvenile and subadult sea turtles and would be influenced by the relatively high number of juvenile turtles that likely comprised our sample size. Further characterization of growth curves in green turtles in Grenada would allow assessment of whether reference intervals need to be separated for immature and mature life stages.

The sample population of green turtles consisted of only one adult female. Whether this sampling bias reflects the aggregate demographic in Grenada is unknown. Grenada currently retains a seven-month legal harvesting season for hardshell turtles which may impact population dynamics (Ross, 1984). Samples need to be obtained for adult female and male turtles in Grenada to examine biochemical analyte concentrations that may be associated with life-stage class and sex including cholesterol, triglyceride, calcium and phosphorus that are associated with egg production in reproductively actively females (Bolten and Bjorndal, 1992).

The 63.3 cm mean CCLn-t for foraging hawksbills sampled in Grenada trended towards those more typically described for subadult turtles (Boulon, 1994; Moncada *et al.,* 1999; Wood *et al*., 2013). The mean CCL is similar to population sizes used for plasma biochemical studies in foraging hawksbills in Florida, USA (Stacy *et al.*, 2023), and Pacific hawksbills sampled in the Galapagos Islands (Muñoz-Pérez *et al.*, 2017). Mean AST activities were substantially higher in hawksbills in Grenada (3.98 μkat/l) compared to those published in Florida (1.88 μkat/l) (Stacy *et al.*, 2023) and the Galapagos Islands (3.25 μkat/l) (Muñoz-Pérez *et al.*, 2017) and reference intervals described in Australia (0.68–2.99 μkat/l) (Whiting *et al.*, 2014) and the Pacific region of El Salvador (0.3–1.23 μkat/l) (Tauer *et al.*, 2017). The cause of the relatively high AST activities in foraging hawksbills compared to other sample populations and foraging green turtles and nesting hawksbills in Grenada is unknown but may represent normal variation for this species. Although plasma AST data was not normally distributed, there were no outliers identified by statistical analysis suggesting that concentrations were representative of the aggregation and not due to a subset of individuals that skewed the data. Aspartate transaminase is an enzyme largely associated with liver and muscle damage (Kerr, 2002) although it is found to be expressed in a wide range of tissues in loggerheads (Anderson *et al.*, 2013). Relatively higher plasma AST has been associated with tourism-based feeding supplementation in green turtles compared to non-supplemented conspecifics (Stewart *et al.*, 2016) suggesting liver disease secondary to inappropriate diets. This is not believed to be the case in Grenada as necropsies of six hawksbills in Grenada have shown gastrointestinal contents to be consistent with diets described in other regions including sponges, algae and invertebrates (Meylan, 1988; Von Brandis *et al.,* 2014; Martínez-Estévez *et al.,* 2022) and histopathology has not indicated lipidosis or hepatocellular alterations (Marancik, unpublished).

Aspartate transaminase is presumably not directly associated with muscle injury or exertion secondary to capture and restraint within this study as enzyme activities are described to reach peak concentrations in 3–4 days post-injury in mammals (Lim, 2020) which would not match the capture and sampling timeframe. Although it is unknown if time to peak activities after tissue injury are similar between mammals and sea turtles, previous studies have not found AST to be increased in green turtles and Kemp’s ridleys up to 3 hours post-capture in gill nets secondary to muscle exertion and damage (Snoddy *et al.*, 2010). Aspartate transaminase activities can be artificially increased in plasma samples due to hemolysis (Koseoglu *et al.*, 2011) although hemolysis was relatively low for this sample population and no association was found between AST and quantified hemolysis when the 18 turtles with low to moderate hemolysis were included in the statistical data set. The high AST activities described appear to result from regional differences and possible derangements need to be more accurately characterized to examine the cause and effect on turtle health.

Analytes associated with metabolism also showed trends in foraging hawksbills that slightly varied compared to previously published data. The mean of 37 g/l for total protein for foraging hawksbills in Grenada trended lower than a 48 g/l mean for foraging Galapagos hawksbills (Muñoz-Pérez *et al.*, 2017) while mean calcium (2.4 mmol/l) and phosphorus (1.9 mmol/l) trended higher and lower, respectively, compared to a mean of 2.00 and 2.58 mmol/l calcium and phosphorus, respectively, in turtles in Florida (Stacy *et al.*, 2023). Hawksbills are omnivorous with diets including a wide variety of benthic invertebrates, algae and other plant material (Martínez-Estévez *et al.*, 2022) which may differ between localities. Differences may also be associated with dissimilarities in turtle size between studies as total protein, phosphorus and calcium concentrations were positively correlated with CCL in our data set. Similar to green turtles, characterizing the age at maturity and an increased sample sizes for adult male and female hawksbill preclude assessing whether reference intervals need to be developed based on life-stage class and sex.

Biochemical analyte results in nesting hawksbills were similar to data available for other aggregations in El Salvador (Tauer *et al.*, 2017), Campeche, Mexico (Salvarani *et al.*, 2018) and the Persian Gulf (Ehsanpour *et al.*, 2015). Mean plasma triglyceride concentrations in Grenadian nesting hawksbills at 2.5 mmol/l were relatively low compared to turtles sampled in Mexico at 5.4 mmol/l (Salvarani *et al.*, 2018) and the Persian Gulf at 4.2 mmol/l (Ehsanpour *et al.*, 2015). Whether this is affected by diet and/or nesting interval similar to leatherbacks (Goldberg *et al.*, 2013; Plot *et al.*, 2013; Perrault and Stacy, 2018) requires serial sampling of hawksbills during nesting season. The sample size for hawksbills was relatively low. Increasing this sample size and defining reference intervals is a goal for future research but sampling is challenging as the remote island location of nesting hawksbills limits access to these aggregations.

Biochemical data from nesting hawksbills were largely similar to foraging hawksbills with the exception of select analytes likely associated with nesting physiology. There were significantly higher cholesterol concentrations, but not triglycerides, and a trend towards higher calcium concentrations in nesting hawksbills that is likely associated with egg production (Mader, 2005; Honarvar *et al.*, 2011). Cholesterol and triglyceride concentrations in nesting hawksbills from this study were significantly lower than nesting leatherbacks suggesting species-specific differences may exist for mobilization of these lipids during nesting. Nesting hawksbills also demonstrated significantly lower AST activities than foraging hawksbills. The opposite might be expected as increased AST may be observed in nesting turtles secondary to muscle exertion from crawling on the beach and nest digging, depending on the timeframe of sampling and the internesting period. Enzyme leakage from muscle exertion was also not supported by similar CK activities in nesting hawksbills compared to foraging hawksbills. No correlations were found between AST and CK activities in either foraging or nesting hawksbills. Plasma CK has been described as being similar for nesting and foraging loggerheads (Deem *et al.*, 2009). Additionally, AST has been described as being negatively correlated with carapace length in Kemp’s ridleys (Perrault *et al.*, 2020) and relatively lower in subadult than adult green turtles (Fong *et al.*, 2010). The larger CCL in nesting versus foraging hawksbills and the lack of correlation between AST and CCL in this study suggests that turtle size was not a major determining factor in the relatively high AST activities in foraging hawksbills. As discussed above, further studies are warranted to examine alterations of AST in hawksbills and the relatively high activities observed for those foraging in Grenada.

It would be advantageous to examine the effect of storage conditions on biochemical analytes commonly evaluated in sea turtles as the remote locations of many turtle aggregates can delay sample processing. Improper blood storage or delays in centrifugation can increase hemolysis of red blood cells (Simundic *et al*., 2020). Experimentally induced hemolysis in nesting leatherback plasma has been shown to affect total protein, mineral and electrolyte concentrations (Stacy *et al.*, 2019). Moderate levels of hemolysis quantified in a subset of leatherbacks, green turtles and hawksbills may have resulted from venipuncture technique as whole blood samples were protected from freezing and centrifugation was performed within hours of sample collection. Although no significant associations were observed between analyte concentrations or enzyme activities when samples with moderate hemolysis index were included in the dataset, they were excluded from reference intervals out of caution.

There are conflicting data regarding the effects of serum and plasma storage on analyte concentrations and enzyme activities. Glucose concentrations have been shown to decrease when stored at room temperature for 48 hours (Kunze *et al.*, 2020) which highlights the importance of maintaining and transporting samples in cold storage in the field, as was performed in this study. Previous studies in mammals have described biochemical analytes to be stable up to 21 days (Quartey *et al.*, 2018), 360 days (Cray *et al.*, 2009) and 740 days (Kolahdoozan *et al.*, 2020) post-centrifugation when stored between −60 and 70°C. Other studies have described accelerated loss of enzyme activities of AST, GGT and CK during the freezing process in freshwater Arrau turtles (*Podocnemis expansa*) (Camargo *et al.*, 2020). Samples in this study were frozen at −80°C for up to 1 year prior to analyte quantification with no freeze–thaw cycles, but possible storage artefacts (e.g. on some enzyme activities) need to be considered as a limitation in this study. These variables need to be examined specifically for sea turtle analytes to assess stability for evaluation.

## Conclusion

This study comprises an important step of defining baseline population health in sea turtles in Grenada to support ongoing conservation efforts. Along with projects involving pathogen surveillance (Edwards *et al.*, 2021; James *et al.*, 2021), reproductive success (Charles *et al*., 2022) and current studies to define population genetics and migration routes of green turtles and hawksbills through haplotype characterization (Westlake *et al.*, 2023), plasma biochemistry is a critical component for developing effective population health surveillance and conservation management. This work also contributes to the collaborative sea turtle medicine programme between Ocean Spirits, Inc. and St. George’s University, School of Veterinary Medicine to rehabilitate injured sea turtles and to return them to the wild. Further sampling of nesting hawksbills is needed to define statistically significant reference intervals which is constrained by innate challenges that accompany sampling sea turtles in uncontrolled field conditions and remote locations. Results obtained for foraging turtles provide a baseline for further sampling to examine the effects of life-stage class on reference intervals. Next steps also include utilizing gel electrophoresis to accurately define albumin and globulin concentrations and to examine protein fractions associated with physiologic and immunological responses (Muller and Brunnberg, 2010; Macrelli *et al.*, 2013). This would contribute to a more robust characterization of sea turtle health in Grenada.
